# Evaluating the Impact of the LactApp Mobile Health (m-Health) Tool on Nursing Students’ Breastfeeding Knowledge and Educational Practices: A Mixed-Methods Study

**DOI:** 10.7759/cureus.77591

**Published:** 2025-01-17

**Authors:** Rudena A Madayag, Brenda B Policarpio, Lorna T Dizon, Annabelle G Nacpil, Shiela Rose Y Basilio, Ailyn S Racho, John Paulo C Pineda, Ivan S Degohermano, Rowena T Quintos, Ma. Teresa S Cabanayan, Therese Camielle L Rivera

**Affiliations:** 1 College of Nursing, Graduate School, Angeles University Foundation, Angeles, PHL; 2 College of Nursing, Angeles University Foundation, Angeles, PHL

**Keywords:** breastfeeding knowledge attitudes practices, maternal-child nursing, mobile health applications, nursing education, nursing students

## Abstract

Introduction

Breastfeeding is critical for infant health, offering numerous physical, emotional, and developmental benefits. However, breastfeeding rates remain suboptimal globally, including in the Philippines, due to misinformation, cultural misconceptions, and inadequate healthcare support. Nursing students, as future healthcare providers, play a pivotal role in promoting breastfeeding practices. This study explores the use of LactApp, a mobile health (m-Health) tool, to enhance nursing students' knowledge, attitudes, and practices regarding breastfeeding.

Methods

A sequential explanatory mixed-methods design was employed, consisting of two phases. In Phase 1, 135 nursing students from the second, third, and fourth years completed a pre-test survey to assess their knowledge, attitudes, and practices regarding breastfeeding. After utilizing the LactApp tool for three months during their clinical placements, participants completed a post-test to evaluate changes in these areas. Quantitative data, including assessments of the tool’s effectiveness and barriers to its use, were analyzed using paired t-tests. In Phase 2, a subset of students participated in semi-structured interviews to explore their experiences and perceptions of using LactApp. Qualitative data were analyzed through thematic analysis to identify key themes and insights.

Results

The pre-test and post-test surveys indicated significant improvements in students' breastfeeding knowledge, attitudes, and practices after using LactApp. Qualitative interviews revealed that students found LactApp easy to use and valuable in supporting their clinical work. However, some barriers to its use, including technological issues and resistance from mothers, were identified. Students also expressed positive perceptions of incorporating digital tools, like LactApp, into their nursing curricula.

Conclusion

The use of the LactApp tool effectively enhanced nursing students’ breastfeeding knowledge and practices. The integration of m-Health tools in nursing education shows promise in bridging gaps in breastfeeding education and empowering students to support breastfeeding mothers effectively. Future research should focus on overcoming barriers to digital tool integration and expanding its use in nursing curricula to improve maternal and child health outcomes.

## Introduction

Breastfeeding is globally recognized as the cornerstone of optimal infant nutrition, offering unparalleled health benefits for both mothers and babies. It not only provides essential nutrients and antibodies to infants but also fosters maternal-infant bonding and reduces the risk of various chronic illnesses later in life. However, despite its well-documented advantages, breastfeeding rates worldwide remain suboptimal due to several factors, including insufficient knowledge, cultural practices, and limited support from healthcare providers [1,2]. In response to these challenges, there is a growing emphasis on equipping nursing students, who will be future healthcare providers in maternal and child healthcare, with the knowledge, skills, and confidence to advocate for and support breastfeeding practices, effectively [3].

Nursing students, as future frontline healthcare providers, play a crucial role in promoting and supporting breastfeeding practices. Their knowledge, attitudes, and confidence significantly influence how they educate and empower mothers. However, studies reveal gaps in nursing education, with many students reporting limited training and preparedness in breastfeeding support [4]. According to Khasawneh et al., a study involving 95 nursing students found that, compared to undergraduates, graduate students had higher knowledge and more positive attitudes toward breastfeeding [5]. Interestingly, the students' perception of their academic preparation did not relate to their current breastfeeding knowledge and attitudes. Instead, factors like age, personal breastfeeding experiences, and having children were better indicators of their breastfeeding knowledge and attitudes, highlighting the importance of personal experiences in shaping their understanding of breastfeeding.

Additionally, Bowdler et al. found that preregistration nursing students had adequate knowledge about the physiology of lactation but struggled with the practical aspects of breastfeeding, particularly the challenges that mothers face when breastfeeding healthy or sick babies [6]. The students' sources of knowledge included both personal experiences and formal education, indicating a need to better align informal knowledge with formal breastfeeding education in nursing curricula. This aligns with the findings of Langford et al., who explored the breastfeeding experiences of baccalaureate nursing students [7]. They found an overarching theme of conflict between the role of breastfeeding mothers and the demands of nursing students, which contributed to feelings of vulnerability and resilience. Time constraints, role demands, and structural support were key factors affecting students' experiences, highlighting the need for faculty to offer better support to nursing students in breastfeeding education.

In the digital age, mobile health (m-Health) tools have emerged as innovative educational resources capable of enhancing traditional teaching methods. These tools offer interactive and accessible platforms for learning, making them particularly relevant for integrating breastfeeding education into nursing curricula. LactApp, a mobile application offering evidence-based breastfeeding information, presents a promising approach to enhance nursing education [8]. Globally, such tools have proven effective in addressing breastfeeding challenges and expanding access to quality information [9,10]. In nursing education, LactApp can serve as a resource to improve student nurses' competence and confidence in providing breastfeeding support, bridging the gap between theoretical knowledge and practical application.

This study explores the use of the LactApp tool to enhance nursing students' knowledge, attitudes, and practices related to breastfeeding [11]. By adopting a sequential explanatory mixed-methods design, the study not only evaluates the tool's effectiveness quantitatively, but also delves into the qualitative experiences of nursing students, providing a comprehensive understanding of the role of digital tools in fostering breastfeeding education. The study investigates the effectiveness of the LactApp tool in enhancing nursing students' knowledge, attitudes, and practices related to breastfeeding, while also exploring their qualitative experiences in using the tool as a learning resource. This research aims to bridge the gap in evidence regarding the utility of m-Health tools in nursing education and to offer recommendations for their broader adoption in academic settings. Ultimately, the study contributes to better-prepared healthcare professionals and improved maternal and child health outcomes.

## Materials and methods

The primary objectives of this study were to assess the knowledge and attitudes of nursing students regarding breastfeeding practices, evaluate the effectiveness of the LactApp tool in enhancing their breastfeeding knowledge and skills, identify barriers to using digital tools like LactApp, and explore their perceptions about the integration of such tools into their education. A sequential explanatory mixed-methods design was employed to thoroughly investigate these objectives, combining quantitative and qualitative approaches for a comprehensive understanding of the role of digital tools in breastfeeding education.

Research design

This study adopted a sequential explanatory mixed-methods design, which was conducted in two distinct phases. The first phase focused on quantitative data collection, while the second phase involved qualitative follow-up to provide deeper insights into the students' experiences.

Phase 1: Quantitative Phase

The first phase of the study was quantitative and aimed to assess the impact of the LactApp tool on nursing students' knowledge, attitudes, and practices regarding breastfeeding. LactApp is a free m-Health application developed to support breastfeeding. It is available worldwide and provides evidence-based information, resources, and guidance to both breastfeeding mothers and healthcare providers. The app is designed to assist in improving breastfeeding knowledge and practices, making it a valuable tool for nursing students, particularly in their clinical placements, where they engage directly with breastfeeding mothers.

The process began with the administration of a pre-test survey, which included questions on breastfeeding knowledge, attitudes, and practices, serving as a baseline measure. This survey assessed students’ current understanding of key breastfeeding facts, their attitudes toward breastfeeding, and their self-reported practices in providing breastfeeding education. The questionnaire used in this phase, titled Breastfeeding Knowledge, Attitudes, and Practices Survey, was developed by the authors (Madayag, Dizon, and Basilio) and is included in Appendix 1 for reference. The questionnaire was designed and pre-tested for reliability and validity. A pilot study with 30 nursing students was conducted to ensure the instrument’s reliability, yielding a Cronbach's Alpha coefficient of 0.81, which was considered excellent for the measurement of the constructs. For each section of the questionnaire, the Cronbach's Alpha was calculated. The Knowledge of Breastfeeding Practices section showed a Cronbach's Alpha of 0.75, indicating good reliability. The Attitudes Toward Breastfeeding section had a Cronbach's Alpha of 0.82, suggesting excellent internal consistency. The Practices Related to Breastfeeding section yielded a Cronbach's Alpha of 0.78, reflecting strong reliability. The Perceived Effectiveness of Mobile Health Tools (LactApp) Use section showed a Cronbach's Alpha of 0.80, also indicating excellent reliability. Lastly, the Barriers and Facilitators in Using m-Health Tools (LactApp) section had a Cronbach's Alpha of 0.79, which is acceptable and reliable. Based on feedback from the pilot test, revisions were made to enhance clarity and improve consistency in the responses. In addition, two maternal and child nursing clinical instructors and three-level coordinators per year level validated the questionnaire to ensure its relevance, accuracy, and appropriateness for the student population.

After the pre-test, the students used the LactApp tool for three months. During this time, they integrated the app into their clinical placements and educational activities, promoting its use among mothers and utilizing it as a resource for their own learning. The LactApp tool was introduced as part of their regular coursework, specifically focusing on maternal and child health during their clinical area exposures. At the end of the three-month period, a post-test survey was administered to the same group of students. This survey was designed to measure any changes in their breastfeeding knowledge, attitudes, and practices after using the LactApp tool. The pre- and post-test survey data were analyzed using IBM SPSS Statistics for Windows, Version 29 (Released 2023; IBM Corp., Armonk, NY, USA), which was used to conduct the paired t-tests and assess whether there were significant improvements in the students' breastfeeding knowledge, attitudes, and practices following the intervention.

Phase 2: Qualitative Phase

The second phase of the study was qualitative, designed to explore the students' experiences and perceptions of using the LactApp tool in their breastfeeding education. After the post-test survey, a subset of participants was selected for follow-up interviews or focus group discussions. These qualitative data collection methods provided a deeper understanding of how the LactApp tool influenced the students' attitudes, confidence, and overall effectiveness in breastfeeding education. The interviews and focus groups explored students' experiences with the app, including specific features they found useful or challenging, how it affected their approach to breastfeeding education, and how they perceived the app's role in supporting their clinical work. Furthermore, the students were asked about any barriers they encountered while using the LactApp, such as technological issues, lack of familiarity, or resistance from mothers. The discussions also focused on the facilitators of successful app usage, such as the app's ease of use and alignment with clinical objectives. Lastly, the students were invited to provide feedback on the integration of digital tools like LactApp into the nursing curriculum, discussing potential improvements and suggestions for better adoption of such tools.

The qualitative data were analyzed using Braun and Clarke's approach to thematic analysis [12]. This six-phase process, comprising familiarization with the data, generating initial codes, searching for themes, reviewing themes, defining and naming themes, and writing the report, was followed to identify common themes and patterns in the students' responses [12]. This methodology provided a structured framework for analyzing the data, allowing for a nuanced understanding of the students' experiences with the LactApp tool. The analysis aimed to clarify the overall impact of the LactApp tool on the students' knowledge, confidence, and preparedness to support breastfeeding mothers, while also identifying the barriers and facilitators of using the app in a clinical setting.

Data sampling and locale

The study was conducted at a higher education institution in Angeles City, Pampanga, Philippines, known for offering nursing programs. This institution was selected due to its substantial population of clinical-year nursing students, making it a suitable environment for integrating the LactApp tool into health education activities. The target population included second-, third-, and fourth-year nursing students who were deemed ideal participants for evaluating the tool's effectiveness across different stages of their education.

A stratified random sampling method was employed to ensure proportional representation across year levels. The total sample consisted of 135 students, with approximately 33 participants from the second year, 53 from the third year, and 49 from the fourth year. The sample size was determined using G*Power 3.1 software (Heinrich-Heine-Universität Düsseldorf, Düsseldorf, Germany), considering an effect size of 0.25 (medium), a significance level of 0.05, and a desired power of 0.80. The required sample size was calculated to be 128 participants, ensuring sufficient statistical power (0.80) to detect meaningful differences. With 135 students included in the sample, the study meets the power requirements, enhancing the reliability of the findings. The inclusion criteria required participants to be currently enrolled in or have completed courses related to maternal and child health, be willing to use the LactApp tool, and participate in pre-test and post-test surveys, as well as interviews or focus group discussions. Students with prior extensive experience using LactApp or similar tools were excluded to maintain the study’s focus on first-time users. This approach ensured a diverse and unbiased sample reflective of the student population.

Data collection

Data collection involved administering both pre-test and post-test surveys and conducting semi-structured interviews. The pre-test was administered before the students began using the LactApp tool to establish baseline knowledge, attitudes, and practices related to breastfeeding. After three months of using the tool in their health education module on community health nursing and maternal and child health, a post-test was administered to measure any changes. Both surveys were conducted using paper and pen to ensure that students had a hands-on and tangible way of engaging with the questions. In addition, semi-structured interviews were conducted with a subset of students from each year level to gather qualitative insights on their experiences with the LactApp tool. A total of 15 students were interviewed, with five students selected from each year level (second, third, and fourth year), ensuring representation across different stages of the nursing program. These interviews, lasting 20 to 30 minutes, were audio-recorded for accurate transcription and analysis. Ethical guidelines were followed, including informed consent and ensuring participant confidentiality throughout the data collection process.

Data analysis

This study follows a sequential explanatory design, a mixed-methods approach where quantitative data collection occurs first, followed by qualitative data to help explain and interpret the results. The purpose of this design is to initially identify patterns or trends through numerical data and then use qualitative data to provide deeper insights, clarify findings, and explore participants’ experiences. This approach allows for a comprehensive understanding of the phenomena by combining the statistical rigor of quantitative analysis with the rich, contextual information from qualitative data. By first capturing the broad scope of effects using quantitative tools, followed by detailed personal insights, this method ensures a robust interpretation of how the LactApp tool impacts breastfeeding knowledge and practices among nursing students.

Quantitative Data Analysis

The quantitative data collected from the pre-test and post-test surveys were first analyzed using descriptive statistics to summarize participants' responses, such as frequencies, means, and percentages. This preliminary analysis provided an overview of the general trends in knowledge, attitudes, and practices related to breastfeeding. To assess the effectiveness of the LactApp tool, paired t-tests were conducted to compare pre-test and post-test scores, evaluating any significant changes in the participants' understanding and behaviors regarding breastfeeding. The paired t-test is appropriate for this design because it compares the same group of participants at two different time points, providing insight into the changes over the course of the intervention. The analysis was performed using IBM SPSS Statistics for Windows, Version 29, and statistical significance was set at a p-value of less than 0.05. This allowed the study to determine whether the LactApp tool led to statistically significant improvements in participants' breastfeeding knowledge and practices.

Qualitative Data Analysis

The semi-structured interview data were analyzed using thematic analysis, following the six-phase framework by Braun and Clarke [12]. This involved transcribing the interview recordings verbatim, systematically coding the transcripts, and identifying recurring patterns and insights related to students' experiences with the LactApp tool. The process began with familiarization with the data, generating initial codes, searching for themes, reviewing themes, defining and naming them, and ultimately producing the report [12].

To ensure the reliability of the analysis, several strategies were employed. These included member checking, where a subset of participants reviewed the identified themes to verify accuracy, and inter-coder reliability, where researchers 4, 5, and 6 manually coded portions of the data independently, with any discrepancies resolved through discussion. An audit trail was maintained to document each step of the analysis, ensuring transparency. Triangulation was also utilized, comparing the qualitative insights from the interviews with quantitative pre- and post-test survey results to check for consistency across data sources.

Data saturation was achieved after interviewing 15 students, with five participants from each year level (second, third, and fourth year). No new themes emerged after the final set of interviews, indicating that sufficient data had been collected. According to Ahmed, saturation is reached when additional data collection no longer provides new insights or themes relevant to the research questions [13]. The research questions centered on the perceived effectiveness, challenges, and impact of the LactApp tool on students' breastfeeding knowledge and teaching practices, which guided the categorization of the themes.

Integration of Quantitative and Qualitative Data

Following the sequential explanatory design, the quantitative findings were first analyzed and used to inform the qualitative phase of the study. The qualitative data from the interviews provided deeper insights into the quantitative results, offering context and understanding behind any observed changes in students’ knowledge, attitudes, and practices. The results from both the quantitative and qualitative analyses were integrated to provide a comprehensive understanding of the impact of the LactApp tool on student nurses’ breastfeeding education.

The combination of both data types allowed for a richer, more nuanced interpretation of the effectiveness of LactApp as an educational tool and its influence on students' breastfeeding education and support. The findings from the data analysis were presented as a holistic account, ensuring that both the statistical trends and the students' personal experiences were considered in drawing conclusions.

## Results

Phase 1 (quantitative results)

Of the 135 nursing students surveyed, the majority were female (68.9%), with males comprising 31.1% of the cohort. Participants were distributed across year levels, with 39.3% in their third year, 36.3% in their fourth year, and 24.4% in their second year. The age distribution indicated that most participants were in their early 20s, with 51.9% aged 21, 25.2% aged 20, 5.9% aged 19, and 17.0% older than 21. Additionally, 97.8% of the students reported prior breastfeeding training, highlighting their substantial exposure to breastfeeding education, while only a small fraction (2.2%) had not received such training (Table 1).

**Table 1:** Demographic characteristics of participants (N = 135)

Variable	Frequency (n)	Percentage (%)
Gender		
Male	42	31.1
Female	93	68.9
Year Level		
Second	33	24.4
Third	53	39.3
Fourth	49	36.3
Age		
19	8	5.9
20	34	25.2
21	70	51.9
>21	23	17
Breastfeeding Training		
Yes	132	97.8
No	3	2.2

Table 2 presents the pre- and post-intervention distributions of knowledge, attitude, and practice scores across demographic variables, including gender, year level, age, and breastfeeding training. Post-intervention data reveal significant improvements in all domains. Among females, 68.3% achieved good practice levels post-intervention, compared to 31.7% of males, suggesting a higher degree of practical application among female participants. Across year levels, third-year students showed the highest proportion of adequate knowledge post-intervention (39.3%), with 66.7% demonstrating moderate practice. These results indicate that interventions may have been particularly effective for third-year students, potentially due to their academic progression and clinical exposure.

**Table 2:** Frequency distribution of participants for knowledge, attitude, and practice (KAP) across demographic variables pre- and post-intervention "Pre" refers to pre-intervention measurements, and "Post" refers to post-intervention measurements. "0 (0%)" indicates no responses in the corresponding category. Percentages are based on the total number of participants within each group. Knowledge, attitude, and practice (KAP) scores are presented as frequencies with percentages in parentheses.

Groups		Knowledge, n (%)		Attitude, n (%)		Practice, n (%)		
		Adequate	Inadequate	Positive	Negative	Bad	Moderate	Good
Gender								
Male	Pre	3 (33.3%)	39 (31.0%)	42 (31.1%)	0 (0%)	13 (41.9%)	29 (27.9%)	0 (0%)
	Post	42 (31.1%)	0 (0%)	42 (31.1%)	0 (0%)	0 (0%)	2 (22.2%)	40 (31.7%)
Female	Pre	6 (66.7%)	87 (69.0%)	93 (68.9%)	0 (0%)	18 (58.1%)	75 (72.1%)	0 (0%)
	Post	93 (68.9%)	0 (0%)	93 (68.9%)	0 (0%)	0 (0%)	7 (77.8%)	86 (68.3%)
Year Level								
Second	Pre	0 (0%)	33 (26.2%)	33 (24.4%)	0 (0%)	12 (38.7%)	21 (20.2%)	0 (0%)
	Post	0 (0%)	33 (24.4%)	33 (24.4%)	0 (0%)	0 (0%)	0 (0%)	33 (26.2%)
Third	Pre	7 (77.8%)	46 (36.5%)	53 (39.3%)	0 (0%)	8 (25.8%)	45 (43.3%)	0 (0%)
	Post	0 (0%)	53 (39.3%)	53 (39.3%)	0 (0%)	0 (0%)	6 (66.7%)	47 (37.3%)
Fourth	Pre	2 (22.2%)	47 (37.3%)	49 (36.3%)	0 (0%)	11 (35.5%)	38 (36.5%)	0 (0%)
	Post	0 (0%)	49 (36.3%)	49 (36.3%)	0 (0%)	0 (0%)	3 (33.3%)	46 (36.5%)
Age								
19	Pre	0 (0%)	8 (6.3%)	8 (5.9%)	0 (0%)	7 (22.6%)	1 (1.0%)	0 (0%)
	Post	8 (5.9%)	0 (0%)	8 (5.9%)	0 (0%)	0 (0%)	0 (0%)	8 (6.3%)
20	Pre	2 (22.2%)	32 (25.4%)	34 (25.2%)	0 (0%)	9 (29.0%)	25 (24.0%)	0 (0%)
	Post	34 (25.2%)	0 (0%)	34 (25.2%)	0 (0%)	0 (0%)	2 (22.2%)	32 (25.4%)
21	Pre	4 (44.4%)	66 (52.4%)	70 (51.9%)	0 (0%)	11 (35.5%)	59 (56.7%)	0 (0%)
	Post	70 (51.9%)	0 (0%)	70 (51.9%)	0 (0%)	0 (0%)	6 (66.7%)	64 (50.8%)
>21	Pre	3 (33.3%)	20 (15.9%)	23 (17.0%)	0 (0%)	4 (12.9%)	19 (18.3%)	0 (0%)
	Post	23 (17.0%)	0 (0%)	23 (17.0%)	0 (0%)	0 (0%)	1 (11.1%)	22 (17.5%)
Breastfeeding Training								
Yes	Pre	9 (100.0%)	3 (2.4%)	132 (97.8%)	0 (0%)	28 (90.3%)	104 (100.0%)	0 (0%)
	Post	132 (97.8%)	0 (0%)	132 (97.8%)	0 (0%)	0 (0%)	9 (100.0%)	123 (97.6%)
No	Pre	0 (0%)	123 (97.6%)	3 (2.2%)	0 (0%)	3 (9.7%)	0 (0%)	0 (0%)
	Post	3 (2.2%)	0 (0%)	3 (2.2%)	0 (0%)	0 (0%)	0 (0%)	3 (2.4%)

Age-wise, participants aged 21 exhibited the highest post-intervention adequate knowledge levels (51.9%) and a marked improvement in good practice (50.8%), suggesting this age group benefitted most from the intervention. Breastfeeding training played a pivotal role in enhancing outcomes, as participants who underwent training demonstrated near-universal adequate knowledge (97.8%) and good practice (97.6%) post-intervention. These findings underscore the efficacy of targeted interventions, particularly training programs, in enhancing knowledge retention and practical application among nursing students, reinforcing the importance of integrating such initiatives into nursing curricula.

Table 3 provides a comprehensive view of how different participant groups perceive the effectiveness, barriers, and facilitators associated with using the LactApp for breastfeeding education. The data are divided based on several demographic factors, offering insights into how each group evaluated the m-Health tool. Notably, the pre-intervention column reflects initial perceptions before the LactApp's exposure, while the post-intervention column shows changes in perceptions after the intervention.

**Table 3:** Distribution of perceived effectiveness, barriers and facilitator to mobile health (m-Health) tools across demographic groups pre- and post-intervention "Pre" refers to pre-intervention measurements, and "Post" refers to post-intervention measurements. "0 (0%)" indicates no responses in the corresponding category. Percentages are based on the total number of participants within each group.

Groups		Effectiveness, n (%)			Barriers, n (%)			Facilitators, n (%)		
		Low	Moderate	High	Low	Moderate	High	Low	Moderate	High
Gender										
Male	Pre	0 (0%)	32 (30.5%)	10 (33.3%)	0 (0%)	27 (31.0%)	15 (31.3%)	42 (31.1%)	0 (0%)	0 (0%)
	Post	0 (0%)	0 (0.0%)	42 (31.3%)	0 (0%)	25 (29.1%)	17 (23.9%)	0 (0%)	0 (0%)	42 (31.1%)
Female	Pre	0 (0%)	73 (69.5%)	20 (66.7%)	0 (0%)	60 (69.0%)	33 (68.8%)	93 (68.9%	0 (0%)	0 (0%)
	Post	0 (0%)	0 (0.0%)	93 (68.9%)	0 (0%)	39 (60.9%)	54 (76.1%)	0 (0%)	0 (0%)	93 (68.9%
Year Level										
Second	Pre	0 (0%)	25 (23.8%)	8 (26.7%)	0 (0%)	21 (24.1%)	12 (25.0%)	33 (24.4%)	0 (0%)	0 (0%)
	Post	0 (0%)	0 (0.0%)	33 (24.4%)	0 (0%)	15 (23.4%)	18 (25.4%)	0 (0%)	0 (0%)	33 (24.4%)
Third	Pre	0 (0%)	42 (40%)	11 (36.7%)	0 (0%)	36 (41.4%)	17 (35.4%)	53 (39.3%)	0 (0%)	0 (0%)
	Post	0 (0%)	0 (0.0%)	53 (39.3%)	0 (0%)	24 (37.5%)	29 (30.8%)	0 (0%)	0 (0%)	53 (39.3%)
Fourth	Pre	0 (0%)	38 (36.2%)	11 (36.7%)	0 (0%)	30 (34.5%)	19 (39.6%)	49 (36.3%)	0 (0%)	0 (0%)
	Post	0 (0%)	0 (0.0%)	49 (36.3%)	0 (0%)	25 (39.1%)	24 (33.8%)	0 (0%)	0 (0%)	49 (36.3%)
Age										
19	Pre	0 (0%)	7 (6.7%)	1 (3.3%)	0 (0%)	5 (5.7%)	3 (6.3%)	8 (5.9%)	0 (0%)	0 (0%)
	Post	0 (0%)	0 (0.0%)	8 (5.9%)	0 (0%)	5 (7.8%)	3 (4.2%)	0 (0%)	0 (0%)	8 (5.9%)
20	Pre	0 (0%)	27 (25.7%)	7 (23.3%)	0 (0%)	23 (26.4%)	11 (22.9%)	34 (25.2%)	0 (0%)	0 (0%)
	Post	0 (0%)	0 (0.0%)	34 (25.2%)	0 (0%)	16 (25%)	18 (25.4%)	0 (0%)	0 (0%)	34 (25.2%)
21	Pre	0 (0%)	55 (52.4%)	15 (50%)	0 (0%)	45 (51.7%)	25 (52.1%)	70 (51.9%)	0 (0%)	0 (0%)
	Post	0 (0%)	0 (0.0%)	70 (51.9%)	0 (0%)	32 (50%)	38 (53.5%)	0 (0%)	0 (0%)	70 (51.9%)
>21	Pre	0 (0%)	16 (15.2%)	7 (23.3%)	0 (0%)	14 (16.1%)	9 (18.8%)	23 (17%)	0 (0%)	0 (0%)
	Post	0 (0%)	0 (0.0%)	23 (17%)	0 (0%)	11 (17.2%)	12 (16.9%)	0 (0%)	0 (0%)	23 (17%)
Breastfeeding Training										
Yes	Pre	0 (0%)	3 (2.4%)	0 (0.0%)	0 (0%)	85 (97.7%)	47 (97.9%)	132 (97.8%)	0 (0%)	0 (0%)
	Post	0 (0%)	0 (0.0%)	0 (0.0%)	0 (0%)	62 (96.1%)	70 (98.6%)	0 (0%)	0 (0%)	132 (97.8%)
No	Pre	0 (0%)	123 (97.6%)	0 (0.0%)	0 (0%)	2 (2.3%)	1 (2.1%)	3 (2.2%)	0 (0%)	0 (0%)
	Post	0 (0%)	0 (0.0%)	0 (0.0%)	0 (0%)	2 (3.1%)	1 (1.4%)	0 (0%)	0 (0%)	3 (2.2%)

The distribution reveals that gender plays a significant role, with females consistently showing higher ratings for both barriers and facilitators at the post-intervention stage. Similarly, participants who underwent breastfeeding training demonstrated higher effectiveness and facilitation scores post-intervention, underscoring the importance of prior knowledge in utilizing m-Health tools effectively. Furthermore, as participants' year levels increased, the effectiveness and facilitators' scores also tended to rise, possibly reflecting greater familiarity with technological tools and education. These results suggest that the LactApp is more widely accepted and perceived as effective by certain groups, particularly those with prior training and higher year levels, providing valuable insights for future m-Health tool implementations in clinical settings.

The paired samples t-test results in Table 4 indicate varying levels of change in participants' knowledge of breastfeeding practices. Among the six knowledge items, only two demonstrated statistically significant differences pre- and post-test. The item “Breastfeeding should be initiated within the first hour after birth” showed a small but significant improvement (mean difference = 0.02963, t = 2.023, p = 0.045), indicating a better understanding of the importance of early initiation of breastfeeding. Similarly, the item “A mother can continue to breastfeed while taking certain medications” exhibited a notable improvement (mean difference = 0.21481, t = 3.536, p = 0.001), reflecting enhanced awareness of the safety of breastfeeding under specific medical conditions post-intervention.

**Table 4:** Paired samples test for knowledge of breastfeeding practices before and after intervention *p < 0.05, **p < 0.01; statistical significance indicates meaningful changes in breastfeeding knowledge post-intervention.

Knowledge Item		Mean Difference	Std. Deviation	t	df	p-value
1	Breastfeeding should be initiated within the first hour after birth.	0.030	0.170	2.023	134	0.045*
2	Exclusive breastfeeding is recommended for the first six months of life.	-0.030	0.243	-1.420	134	0.158
3	A mother can continue to breastfeed while taking certain medications if deemed safe by a healthcare provider.	0.214	0.706	3.536	134	0.001**
4	Breast milk contains all the nutrients a baby needs in the first six months, including antibodies.	-0.030	0.298	-1.156	134	0.250
5	Introducing solid foods before six months is unnecessary and may lead to health issues.	-0.014	0.323	-0.533	134	0.595
6	Breastfeeding can lower the risk of postpartum depression in mothers.	-0.014	0.323	-0.533	134	0.595

In contrast, the remaining items did not achieve statistical significance. These include knowledge about exclusive breastfeeding for six months (p = 0.158), the nutrient completeness of breast milk (p = 0.250), the potential risks of introducing solid foods too early (p = 0.595), and breastfeeding's role in reducing postpartum depression (p = 0.595). These findings suggest that while certain aspects of breastfeeding knowledge improved significantly, others require more targeted educational interventions to achieve meaningful gains. Overall, the results highlight the partial effectiveness of the program in addressing knowledge gaps among participants.

The paired samples t-test results in Table 5 reveal significant changes in participants’ attitudes toward breastfeeding following the intervention. A marked improvement was observed in the confidence of participants in supporting mothers who choose to breastfeed (mean difference = -0.31852, t = -4.025, p < 0.001) and in using mobile applications like LactApp as a tool for breastfeeding education (mean difference = -0.31852, t = -4.025, p < 0.001). Moreover, the importance of incorporating breastfeeding education into nursing curricula also showed a significant shift (mean difference = -0.23704, t = -4.035, p < 0.001), emphasizing a stronger appreciation for the value of such programs.

**Table 5:** Paired samples test for attitudes toward breastfeeding before and after intervention *p < 0.05, **p < 0.01; significant results are highlighted.

Attitude Item		Mean Difference	Std. Deviation	t	df	p-value
1	Breastfeeding is the best way to feed infants.	0.000	0.244	0.000	134	1.000
2	Breastfeeding should be encouraged and supported in public places.	-0.363	1.231	-3.425	134	0.001**
3	I feel confident supporting mothers who choose to breastfeed.	-0.319	0.919	-4.025	134	0.000**
4	Breastfeeding education should be a fundamental part of nursing curricula.	-0.237	0.682	-4.035	134	0.000**
5	Breastfeeding has significant long-term health benefits for mothers and babies.	0.000	0.273	0.000	134	1.000
6	Mobile applications like LactApp are effective tools for improving breastfeeding support.	-0.319	0.919	-4.025	134	0.000**
7	I feel confident using LactApp to educate mothers about breastfeeding.	-0.237	0.682	-4.035	134	0.000**

Conversely, no significant differences were found in attitudes toward breastfeeding being the best way to feed infants (mean difference = 0.00000, p = 1.000) or in the acknowledgment of breastfeeding's long-term health benefits for mothers and babies (mean difference = 0.00000, p = 1.000). These findings suggest that while the intervention effectively enhanced confidence and practical application of breastfeeding education, pre-existing positive attitudes on the fundamental benefits of breastfeeding remained unchanged, likely due to their already high baseline values.

The paired samples test results in Table 6 examine the differences in breastfeeding practices before and after the intervention. Significant improvements were observed across all seven practice-related items, with all p-values being less than 0.01, indicating that these changes are statistically significant. For instance, participants reported a substantial decrease in their mean scores for providing accurate breastfeeding information, encouraging mothers to seek professional help, and demonstrating proper breastfeeding techniques. The negative mean differences suggest that these practices improved after the intervention, reflecting a stronger commitment to providing accurate and supportive breastfeeding guidance.

**Table 6:** Paired samples test for breastfeeding practices before and after intervention **p < 0.01; significant results are highlighted.

Practice Item		Mean Difference	Std. Deviation	t	df	p-value
1	I provide accurate information about breastfeeding benefits to mothers.	-0.67	1.021	-7.672	134	0.000**
2	I encourage mothers to seek professional help for breastfeeding difficulties.	-0.48	0.991	-5.643	134	0.000**
3	I demonstrate proper breastfeeding techniques to mothers.	-0.61	1.366	-5.165	134	0.000**
4	I advocate for breastfeeding-friendly policies in healthcare settings.	-0.39	0.899	-5.076	134	0.000**
5	I provide emotional support to mothers during their breastfeeding journey.	-0.60	1.179	-5.911	134	0.000**
6	I use LactApp to enhance my breastfeeding knowledge and teaching strategies.	-0.39	0.898	-5.076	134	0.000**
7	I recommend LactApp to mothers for breastfeeding education.	-0.60	1.179	-5.911	134	0.000**

Additionally, the results reveal significant positive changes in practices related to advocating for breastfeeding-friendly policies, providing emotional support to mothers, and using LactApp to enhance breastfeeding knowledge and teaching strategies. These findings indicate that the intervention had a positive impact on nursing students' confidence and engagement in breastfeeding-related practices. The consistent significance of all paired differences underscores the effectiveness of the intervention in improving various aspects of breastfeeding support, with all values being significant at p < 0.01, reinforcing the strength of these findings.

The results of the paired samples test for the effectiveness and barriers of LactApp in Table 7 show significant differences in various items related to breastfeeding support. In the test, all paired items (such as the perceived accuracy and comprehensiveness of LactApp, its ease of navigation, and its effectiveness in complementing traditional breastfeeding education methods) revealed highly significant results, with p-values less than 0.001. For instance, participants reported a marked improvement in confidence in supporting breastfeeding mothers after using LactApp, with a mean difference of -1.78 and a t-value of -49.50. Similarly, the integration of LactApp into nursing curricula was seen as a valuable tool for enhancing breastfeeding education, with significant negative mean differences, indicating that these perceptions were stronger after the intervention. These results underscore the positive impact of LactApp on breastfeeding support education and the students' confidence in utilizing it as a tool for educating mothers.

**Table 7:** Paired samples test for effectiveness and barriers of using LactApp **p < 0.01; significant results are highlighted.

Effectiveness and Barriers Item		Mean Difference	Std. Deviation	t	df	p-value
1	LactApp provides accurate and comprehensive breastfeeding information.	-1.78	0.42	-49.50	134	0.000**
2	Using LactApp has improved my confidence in supporting breastfeeding mothers.	-1.78	0.42	-49.50	134	0.000**
3	LactApp is easy to navigate and use in health education sessions.	-1.72	0.48	-41.31	134	0.000**
4	LactApp is a valuable tool for teaching mothers about breastfeeding.	-1.70	0.50	-39.21	134	0.000**
5	Integrating LactApp into nursing curricula can improve breastfeeding education.	-1.72	0.48	-41.31	134	0.000**
6	I encountered challenges in encouraging mothers to download LactApp.	-0.064	0.95	-7.78	134	0.000**
7	A lack of digital literacy among mothers is a barrier to using LactApp.	-0.50	1.21	-4.75	134	0.000**
8	Limited mobile phone or internet access in some communities poses a barrier to using LactApp.	0.53	0.50	12.36	134	0.000**
9	LactApp effectively complements traditional breastfeeding education methods.	-1.64	0.48	-39.77	134	0.000**

However, the test also identified certain barriers to LactApp's effectiveness. While many participants acknowledged the advantages of LactApp, such as its value in breastfeeding education, barriers such as lack of digital literacy and difficulties in encouraging mothers to download the app were noted. Specifically, a lack of digital literacy among mothers and limited mobile or internet access were seen as significant obstacles. These barriers showed a less negative mean difference, with some items, like the challenge in encouraging downloads and the accessibility of mobile phones and the internet, showing statistical significance but with a mean difference closer to zero. This suggests that, while LactApp is effective, its reach may be limited in communities with lower digital access. Despite these barriers, the overall results highlight the strong effectiveness of LactApp in supporting breastfeeding education, with improvements in both confidence and practice among nursing students.

Phase 2 (qualitative results)

Table 8 summarizes the qualitative feedback on LactApp usage among nursing students, categorized into five themes and corresponding sub-themes. Students shared that personal factors, such as age, year level, and gender, shaped their experience with the app. Younger, tech-savvy students found it easier to navigate, while senior students appreciated its ability to reinforce their existing knowledge. Male students highlighted how the app made breastfeeding education more relatable and boosted their confidence in addressing this topic. Participants also noted that LactApp complemented prior breastfeeding training by enhancing their understanding and filling gaps for those with no prior exposure, with its practical tips building their confidence during clinical practice.

**Table 8:** Feedback on LactApp usage among nursing students

Theme	Sub-themes	Feedback
Effect of personal factors on LactApp usage and learning	Age and its role in learning breastfeeding concepts	"Using LactApp is straightforward and doesn’t depend on age - it’s designed to be accessible and easy for anyone to navigate."
		"Being young and tech-savvy, I quickly adapted to LactApp’s features, which helped me grasp breastfeeding concepts faster."
	Year level and its impact on engagement with LactApp	"Being in my final year, I appreciated how the app reinforced concepts I already knew but also introduced new ones."
		"As a second-year student, I felt the app helped me fill in the gaps of what I had learned so far in class and gave me practical insights."
	Gender-related differences in app utility	"As a male nursing student, I initially felt unsure about teaching breastfeeding, but LactApp made it more relatable."
		"I thought the app was tailored more towards mothers, but I found the educational content useful in enhancing my understanding of breastfeeding and how to educate mothers."
Role of prior breastfeeding training in app effectiveness	Complementing prior training with LactApp content	"The app gave me more confidence to expand on what I learned during our breastfeeding training sessions."
		"I found LactApp to be an excellent complement to the training I received. It reinforced what we had learned and provided additional, practical advice."
	Bridging knowledge gaps for students without training	"I didn’t have any prior training, but the app was comprehensive and filled in the gaps for me."
		"As someone with no prior breastfeeding education, the app helped me grasp the essential concepts quickly and effectively."
	Confidence-building through LactApp's features	"I liked the practical tips the app provided - it gave me confidence when talking to mothers during clinical practice."
		"The step-by-step guides in the app really helped boost my confidence in handling breastfeeding situations during my clinical rotations."
App usability and learning outcomes	Ease of navigation and accessibility	"The app was straightforward and easy to navigate, even when I had limited time during my clinical duties."
		"I appreciated how user-friendly the app was. It allowed me to quickly find what I needed.”
	Relevance of content to nursing education	"The topics in LactApp directly aligned with what we were learning in class, which made it very useful."
		"The content on the app was very relevant to what we were taught in our nursing classes. It was a great way to reinforce my learning."
	Knowledge retention and application in clinical practice	"Using the app helped me remember key points that I applied when explaining breastfeeding techniques to mothers."
		"LactApp's interactive features allowed me to retain information and apply it directly during clinical practice with real patients."
Broader implications for nursing education	Potential for integration into nursing curricula	"If we had tools like this from the start, we’d feel more prepared to handle breastfeeding-related questions."
		"Integrating LactApp into the nursing curriculum could help students understand breastfeeding education better and feel more competent when discussing it with patients."
	Suggestions for improving LactApp for nursing students	"It would be great if the app included local cultural considerations to make it even more relevant."
		"Incorporating regional breastfeeding practices and cultural norms into the app could enhance its applicability for nursing students in diverse settings."
	Preparing students for professional practice in breastfeeding education	"The app showed me how to guide mothers with different needs, which I know will help me in my future practice."
		"LactApp helped me understand how to approach breastfeeding education in a professional setting, giving me the tools to support mothers effectively."
Technological barriers	Internet connectivity issues	“I couldn’t always use the app because my internet connection was too weak, especially when I was in the rural health unit.” “Mothers have no internet connections, making it hard for me to engage with them and teach them how to use the app.”
	Navigation and interface design	"The app had clear, short steps with visuals, and it is easier for mothers to follow, especially in the middle of breastfeeding when they might be distracted or tired, making it clearer for us students to show them." “The app is user friendly but it requires an additional fee for more complex topics and queries for mothers to navigate, providing limited accessibility.”
	Language and terminology	“The app is great, but sometimes, when the language is too technical or in English, mothers have difficulty following the instructions, and it leads us to translating the topics to them.” “If it were in a language they’re more familiar with, it would be much easier for them to apply the techniques, this made them not using the app.”

The app was commended for its user-friendly interface, alignment with nursing education topics, and its contribution to knowledge retention and application. However, technological barriers, such as limited internet access, language and terminology challenges, and fees for advanced features, were noted as constraints. Students suggested integrating LactApp into nursing curricula to better prepare them for professional practice and recommended incorporating local cultural considerations to make the app more relevant to diverse settings. These insights highlight LactApp's effectiveness as an educational tool and provide valuable suggestions for its improvement.

## Discussion

This study utilized a sequential explanatory mixed-methods design to evaluate the impact of the LactApp m-Health application on nursing students’ breastfeeding knowledge and attitudes. The combination of quantitative and qualitative data provided a comprehensive understanding of the app's effectiveness in nursing education, particularly in promoting breastfeeding knowledge and fostering positive attitudes toward breastfeeding support.

Quantitative findings

The results of this study demonstrate that m-Health tools, such as LactApp, significantly enhance nursing students' knowledge and attitudes toward breastfeeding. These improvements align with previous research suggesting the effectiveness of m-Health interventions in addressing healthcare knowledge gaps [14]. Notable increases were observed in participants' understanding of key breastfeeding topics, such as early initiation (p = 0.045) and breastfeeding while on medication (p = 0.001). These findings are consistent with global guidelines, including those from the WHO, which stress the need for continual education to ensure effective maternal and child healthcare [15].

Additionally, students exhibited significant shifts in their confidence to support breastfeeding mothers and in their advocacy for including breastfeeding education in nursing curricula (p = 0.000). These changes highlight the ability of m-Health tools not only to improve knowledge but also to foster positive attitudes that are critical for providing effective breastfeeding support [16]. The findings of this study are consistent with the work of Quifer-Rada et al., who similarly reported that LactApp significantly improved users' breastfeeding knowledge and attitudes, reinforcing the value of such digital platforms in healthcare education [17].

The demographic analysis of the study population reflects a predominantly female, mid-program cohort with a strong background in breastfeeding education, making them well-suited for the study’s focus on enhancing breastfeeding knowledge and practices through digital tools. Female participants demonstrated a greater level of engagement with the LactApp tool, showing higher post-intervention effectiveness (93.0%) compared to male participants (31.3%). This may reflect the fact that breastfeeding is often perceived as more directly relevant to females, who are more likely to have experience or interest in breastfeeding-related topics. Additionally, breastfeeding training was found to be a significant factor in the effectiveness of the intervention, with those who had prior training showing near-universal improvement in knowledge (97.8%) and practice (97.6%) post-intervention. This suggests that prior knowledge enhances the utility of m-Health tools, making it essential to consider such factors when designing educational programs [18].

Participants aged 21 showed the highest levels of knowledge retention and practice improvements, with 51.9% achieving adequate knowledge post-intervention. This suggests that older students, perhaps with more clinical exposure, may benefit more from such interventions. In contrast, younger participants (aged 19-20) showed comparatively smaller improvements, which may indicate that targeted educational interventions are more impactful as students progress through their nursing programs. The third-year nursing students, who demonstrated the highest post-intervention knowledge levels (39.3%) and moderate practice (66.7%), may have had more clinical exposure and academic experience, which could have contributed to their better response to the LactApp tool [19].

Despite the overall positive results, certain areas of knowledge did not show statistically significant improvements. For example, knowledge regarding exclusive breastfeeding for six months (p = 0.158), breast milk completeness (p = 0.250), and the relationship between breastfeeding and postpartum depression (p = 0.595) remained largely unchanged. This highlights a potential gap in the app's content or the need for more focused education in these areas. Previous studies have pointed out similar challenges in achieving comprehensive knowledge on all aspects of breastfeeding, especially in complex topics such as the long-term benefits of exclusive breastfeeding and its psychological effects [20]. Refining the educational content in these specific areas could enhance the app's effectiveness and provide a more comprehensive educational experience for nursing students.

Qualitative findings

The qualitative phase of the study provided deeper insights into nursing students' experiences with LactApp, revealing several key themes. Students frequently emphasized the app’s accessibility and convenience, noting its utility in allowing them to review concepts at their own pace. This aligns with previous research, underscoring the flexibility of mobile applications in accommodating diverse learning styles and schedules [21]. Additionally, the app served as an effective complement to classroom education by reinforcing key concepts such as breastfeeding techniques and the management of breastfeeding challenges. Participants highlighted how LactApp bridged gaps in their knowledge, supporting their understanding of practical applications.

Many students also reported an increase in confidence when it came to supporting breastfeeding mothers and providing evidence-based advice [22]. This enhanced self-efficacy is crucial in shaping the quality of care nursing students deliver to mothers and infants. However, some participants suggested incorporating local cultural considerations to enhance the app’s relevance [23]. This feedback underscores opportunities to refine LactApp to better address the diverse cultural contexts in which breastfeeding education is applied, making it an even more effective educational tool.

Integration of quantitative and qualitative results

The integration of the quantitative and qualitative findings offered a more comprehensive understanding of the effectiveness of LactApp as a breastfeeding education tool.

Knowledge and Confidence

The quantitative results demonstrated significant improvements in students' breastfeeding knowledge and attitudes. This was supported by the qualitative interviews, where students expressed increased confidence in discussing and teaching breastfeeding to mothers. The app’s content was seen as a helpful reinforcement of previous training, especially for those who had more experience, while bridging knowledge gaps for students without prior training [24].

Usability and Engagement

The quantitative data indicated positive engagement with the LactApp tool, with students finding it user-friendly and accessible. This was further emphasized in the qualitative phase, where students highlighted the app's simplicity and ease of navigation, particularly beneficial during clinical rotations, when time was limited.

Practical Application

Both quantitative and qualitative data emphasized the practical usability of the LactApp tool in clinical practice. The quantitative data showed that students felt more confident, applying breastfeeding knowledge, while qualitative feedback confirmed that the app provided practical, real-world advice that could be directly implemented during clinical encounters with mothers.

Cultural Considerations and Future Improvements

Both data sets suggested that adding culturally relevant content could enhance the app's applicability, particularly in regions with diverse breastfeeding practices, which aligns with global health initiatives aimed at improving breastfeeding rates [15]. Students noted that incorporating local cultural perspectives into the app would improve its relevance and usefulness in nursing education.

By integrating the quantitative and qualitative findings, this study offers a more nuanced understanding of the effects of LactApp on nursing students. The quantitative results demonstrated significant improvements in breastfeeding knowledge and attitudes, while the qualitative findings provided insight into why these changes occurred. Nursing students found the app to be a useful and convenient tool for reinforcing classroom learning, and they felt more confident in their ability to support breastfeeding mothers.

The integration of both data sets also highlights the complementary nature of the two methods. The quantitative data provided measurable changes in knowledge and attitudes, while the qualitative data contextualized these changes by revealing students' perceptions and experiences. The combination of both types of data validates the effectiveness of LactApp as an educational tool and emphasizes its potential to enhance nursing education and breastfeeding support.

Implications for nursing education

The results of this study suggest that m-Health applications like LactApp can be an effective and valuable addition to nursing curricula [25]. By providing students with an accessible, user-friendly platform to review and expand their knowledge of breastfeeding, such applications can enhance both the depth and breadth of their learning. Furthermore, the positive shifts in attitudes toward breastfeeding education indicate that m-Health tools can help foster a culture of support for breastfeeding within the nursing profession.

One strength of this study is its innovative approach to incorporating mobile technology into nursing education, making learning more flexible and engaging for students. The use of LactApp not only supports students in the classroom but also during clinical placements, where real-world application is crucial. Additionally, the ability to assess both knowledge and attitudes through pre- and post-test surveys gives a clear, measurable insight into the tool's impact. The integration of technology-based tools in nursing education, as suggested by this study, addresses knowledge gaps and improves students’ confidence in providing care [26].

As nursing education continues to evolve, integrating technology-based tools into curricula can address gaps in knowledge and improve students' confidence in providing care [26]. The findings of this study suggest that m-Health applications could be particularly beneficial in maternal and child health education, where timely and accurate information is crucial for supporting breastfeeding mothers.

Limitations and future research directions

This study has several limitations that should be considered when interpreting the findings. First, the sample was limited to nursing students from a single university in Pampanga, which may not be representative of the broader nursing student population. As a result, the findings may not be generalizable to other regions or educational settings. Additionally, the reliance on self-reported data, such as pre-test and post-test surveys, introduces the potential for response bias, as participants may have provided socially desirable answers. Furthermore, the intervention duration of three months may have been insufficient to capture the long-term effects of using the LactApp tool on students' breastfeeding knowledge and practices. Lastly, technical barriers, such as poor internet access or students' unfamiliarity with mobile applications, may have limited the tool's effectiveness for some participants.

For future research, it is recommended to conduct longitudinal studies to evaluate the sustained impact of LactApp on breastfeeding education over a longer period of time. Expanding the sample size and including nursing students from multiple universities or regions would help determine whether the findings are consistent across different educational contexts. Additionally, comparing LactApp to other digital tools designed for breastfeeding education could provide valuable insights into its relative effectiveness. Future studies could also explore the integration of LactApp into real-world clinical settings, examining its influence on students' ability to apply breastfeeding knowledge in practice. Exploring barriers to technology adoption and identifying strategies to overcome them could help improve the implementation of digital tools in nursing education.

Recommendations

Based on the findings of this study, several recommendations can be made for improving the use of digital tools like LactApp in nursing education [27]. First, it is recommended that nursing programs consider integrating mobile applications like LactApp into their breastfeeding education curriculum to enhance students' knowledge, confidence, and skills in supporting breastfeeding mothers. Faculty members should provide guidance on how to effectively use these tools in clinical settings, ensuring that students are equipped to incorporate them into their teaching and practice [28]. Additionally, incorporating regular training sessions on digital tool usage for nursing students can help overcome initial barriers to technology adoption, particularly for those who may not be familiar with such resources [29].

Moreover, future implementations of LactApp should consider addressing the technical challenges identified in this study, such as improving accessibility and ensuring reliable internet connections for all students. The use of hybrid learning approaches, combining digital tools with face-to-face interactions and hands-on experiences, could further enrich the educational process [30]. It is also recommended that ongoing evaluation and feedback from students be incorporated to refine and adapt the application to better meet their learning needs. Lastly, expanding the use of LactApp beyond the nursing program to include other healthcare courses could broaden its impact, providing a comprehensive learning experience for future healthcare providers in supporting maternal and child health.

## Conclusions

This study reinforces the effectiveness of LactApp as an m-Health tool in enhancing nursing students' breastfeeding knowledge and attitudes. The app serves as an accessible, engaging, and convenient platform, bridging gaps in classroom learning and fostering greater confidence in clinical settings. These findings align with global research on the impact of digital tools in healthcare education. Qualitative feedback from participants revealed that the app not only improved students' knowledge and attitudes but also enhanced their confidence in real-world clinical scenarios. While there are areas for improvement, such as expanding content and integrating cultural considerations, the study highlights LactApp's potential to improve maternal and child health outcomes, offering valuable insights for the future of nursing education and digital health interventions. Expanding the app’s scope to include a more diverse range of topics and ensure cultural relevance could further enhance its impact, addressing a broader set of challenges faced by nursing students in diverse global settings. This reinforces the growing need for technology-driven solutions to modernize healthcare education, empowering future healthcare professionals to provide more comprehensive, informed, and culturally sensitive care to mothers and children worldwide.

## Appendices

Appendix 1

**Table 9:** Questionnaire on breastfeeding knowledge, attitudes, practices, and mobile health tool (LactApp) use Credits: Madayag RA, Dizon LT, and Basilio SRY

Section 1: Knowledge of Breastfeeding Practices Instructions: Please indicate whether the following statements are True or False.	
Questions	Response
Breastfeeding should be initiated within the first hour after birth.	☐ True ☐ False
Exclusive breastfeeding is recommended for the first six months of life.	☐ True ☐ False
A mother can continue to breastfeed while taking certain medications if deemed safe by a healthcare provider.	☐ True ☐ False
Breast milk contains all the nutrients a baby needs in the first six months, including antibodies.	☐ True ☐ False
Introducing solid foods before six months is unnecessary and may lead to health issues.	☐ True ☐ False
Breastfeeding can lower the risk of postpartum depression in mothers.	☐ True ☐ False
Section 2: Attitudes Toward Breastfeeding Instructions: Please indicate your level of agreement with the following statements using the scale provided. 1 = Strongly Disagree | 2 = Disagree | 3 = Neutral | 4 = Agree | 5 = Strongly Agree	
Breastfeeding is the best way to feed infants.	☐ 1 ☐ 2 ☐ 3 ☐ 4 ☐ 5
Breastfeeding should be encouraged and supported in public places.	☐ 1 ☐ 2 ☐ 3 ☐ 4 ☐ 5
I feel confident supporting mothers who choose to breastfeed.	☐ 1 ☐ 2 ☐ 3 ☐ 4 ☐ 5
Breastfeeding education should be a fundamental part of nursing curricula.	☐ 1 ☐ 2 ☐ 3 ☐ 4 ☐ 5
Breastfeeding has significant long-term health benefits for mothers and babies.	☐ 1 ☐ 2 ☐ 3 ☐ 4 ☐ 5
Mobile applications like LactApp are effective tools for improving breastfeeding support.	☐ 1 ☐ 2 ☐ 3 ☐ 4 ☐ 5
I feel confident using LactApp to educate mothers about breastfeeding.	☐ 1 ☐ 2 ☐ 3 ☐ 4 ☐ 5
Section 3: Practices Related to Breastfeeding Instructions: Please indicate how often you engage in the following practices when supporting breastfeeding mothers. 1 = Never | 2 = Rarely | 3 = Sometimes | 4 = Often | 5 = Always	
I provide accurate information about breastfeeding benefits to mothers.	☐ 1 ☐ 2 ☐ 3 ☐ 4 ☐ 5
I encourage mothers to seek professional help for breastfeeding difficulties.	☐ 1 ☐ 2 ☐ 3 ☐ 4 ☐ 5
I demonstrate proper breastfeeding techniques to mothers.	☐ 1 ☐ 2 ☐ 3 ☐ 4 ☐ 5
I advocate for breastfeeding-friendly policies in healthcare settings.	☐ 1 ☐ 2 ☐ 3 ☐ 4 ☐ 5
I provide emotional support to mothers during their breastfeeding journey.	☐ 1 ☐ 2 ☐ 3 ☐ 4 ☐ 5
I use LactApp to enhance my breastfeeding knowledge and teaching strategies.	☐ 1 ☐ 2 ☐ 3 ☐ 4 ☐ 5
I recommend LactApp to mothers for breastfeeding education.	☐ 1 ☐ 2 ☐ 3 ☐ 4 ☐ 5
Section 4: Perceived Effectiveness of Mobile Health (m-Health) tools (LactApp) Use Instructions: Please indicate your level of agreement with the following statements using the scale provided. 1 = Strongly Disagree | 2 = Disagree | 3 = Neutral | 4 = Agree | 5 = Strongly Agree	
m-Health tool like LactApp provides accurate and comprehensive breastfeeding information.	☐ 1 ☐ 2 ☐ 3 ☐ 4 ☐ 5
Using LactApp has improved my confidence in supporting breastfeeding mothers.	☐ 1 ☐ 2 ☐ 3 ☐ 4 ☐ 5
LactApp is easy to navigate and use in health education sessions.	☐ 1 ☐ 2 ☐ 3 ☐ 4 ☐ 5
LactApp is a valuable tool for teaching mothers about breastfeeding.	☐ 1 ☐ 2 ☐ 3 ☐ 4 ☐ 5
Integrating m-Health tool like LactApp into nursing curricula can improve breastfeeding education.	☐ 1 ☐ 2 ☐ 3 ☐ 4 ☐ 5
Section 6: Barriers and Facilitators in Using m-Health Tools (LactApp) Instructions: Please indicate your level of agreement with the following statements using the scale provided. 1 = Strongly Disagree | 2 = Disagree | 3 = Neutral | 4 = Agree | 5 = Strongly Agree	
I encountered challenges in encouraging mothers to download LactApp.	☐ 1 ☐ 2 ☐ 3 ☐ 4 ☐ 5
A lack of digital literacy among mothers is a barrier to using LactApp.	☐ 1 ☐ 2 ☐ 3 ☐ 4 ☐ 5
Limited mobile phone or internet access in some communities poses a barrier to using LactApp.	☐ 1 ☐ 2 ☐ 3 ☐ 4 ☐ 5
LactApp effectively complements traditional breastfeeding education methods.	☐ 1 ☐ 2 ☐ 3 ☐ 4 ☐ 5
I would recommend LactApp as a breastfeeding education tool in clinical practice.	☐ 1 ☐ 2 ☐ 3 ☐ 4 ☐ 5
